# Mechanical properties and durability of asphalt-concrete composites enhanced with recycled concrete and porcelain aggregates

**DOI:** 10.1016/j.mex.2025.103296

**Published:** 2025-03-29

**Authors:** Waleed A. Hamad, Ganjeena J. Khoshnaw

**Affiliations:** a Construction and Material Technology Engineering Department, Erbil Technology College, Erbil Polytechnic University, Erbil, Iraq; b Road Construction Department, Erbil Technology College, Erbil Polytechnic University, Erbil, Iraq; c Civil Department, Faculty of Engineering, Tishk International University, Erbil, Iraq

**Keywords:** Mechanical property, Abrasion resistance, Rutting resistance, Porcelain-Modified Asphalt Concrete (PMAC) Method

## Abstract

The performance of asphalt–concrete composites in engineering projects is influenced by various factors. Researchers have explored modifying these properties by incorporating recycled materials, often using recycled concrete aggregates in asphalt mixtures with acceptable results. This study investigates whether replacing recycled concrete aggregates with waste porcelain in different amounts could make the concrete stronger, last longer, and improve the environment.

• The methodology involved evaluating optimized aggregate proportions through wheel tracking and Böhme abrasion tests while ensuring compliance with Marshall's stability requirements.
• This changed mix had a 6 % higher bulk specific gravity (2.24), a better Marshall flow (from 3.5 to 4.6 mm), and a 5 % higher stability (peaking at 33 kN).
• The 25 % porcelain replacement exhibited the highest abrasion resistance and extended service life, though it also increased susceptibility to temperature variations.

The results show that adding porcelain waste to asphalt mixtures can help make pavement solutions that are durable, cost-effective, and good for the environment. This research supports sustainable infrastructure development by demonstrating an innovative approach to waste utilization in road construction while maintaining high mechanical performance standards.

## Graphical abstract

**Figure:**
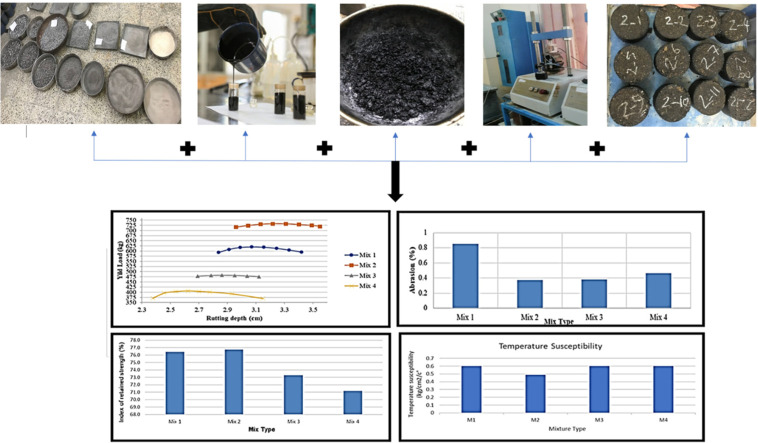

Specifications table

**Table:** 

Subject area:	Engineering
More specific subject area:	A narrower subject area for this article would be the use of recycled porcelain waste as a partial replacement for recycled concrete aggregates (RCA) in asphalt-concrete mixtures.
Name of your method:	Porcelain-Modified Asphalt Concrete (PMAC) Method
Name and reference of original method:	Recycled Concrete Aggregate (RCA) in Asphalt-Concrete Mixtures.Key Reference: • Authors: Behera, M., Bhattacharyya, S.K., Minocha, A.K., Deoliya, R., & Maiti, S. • Title: ``Recycled aggregate from C&D waste & its use in concrete – A breakthrough towards sustainability in the construction sector: A review.'' • Journal: Construction and Building Materials, vol. 68, 2014, pp. 501–516. • DOI: 10.1016/j.conbuildmat.2014.07.003 This review covers the performance of recycled concrete aggregates (RCA) in various construction applications, including their use in concrete and asphalt mixtures.
Resource availability:	To reproduce the Porcelain-Modified Asphalt Concrete (PMAC) Method, the following resources are necessary: 1. Equipment Asphalt Mixing Plant: A standard laboratory or industrial asphalt mixing plant for preparing asphalt-concrete mixtures. Marshall Stability Testing Apparatus: To evaluate the stability and flow properties of the asphalt mixtures according to the Marshall method. Wheel Tracking Machine: For conducting rutting resistance tests on the modified asphalt mixtures. Bohme Abrasion Testing Machine: To measure the abrasion resistance of the porcelain-modified asphalt. Compactor: A mechanical compactor or gyratory compactor to mold asphalt concrete specimens. 2. Materials Porcelain Waste: Sourced from waste ceramic or porcelain industries or recycling centers, this material is used to replace recycled concrete aggregates. Recycled Concrete Aggregates (RCA): Commonly available from construction and demolition waste. Asphalt Binder: A typical bitumen binder used in asphalt concrete mixes. 3. Standards and Guidelines Marshall Test Guidelines: Follow standards like ASTM D6927 (Standard Test Method for Marshall Stability and Flow of Asphalt Mixtures). Bohme Abrasion Test Guidelines: Refer to DIN 52,108 (Testing of Floor Coverings). Wheel Tracking Test Guidelines: Use standards like ASTM D6373 (Specification for Performance-Graded Asphalt Binder). 4. Data and Software Data Collection: Software or manual recording of results from testing equipment (Marshall test, wheel tracking test, Bohme abrasion test). Data Analysis: Software such as Microsoft Excel, MATLAB, or other statistical analysis tools for analyzing the mechanical properties and performance of the asphalt mixtures.

## Background

Asphalt pavement technology has evolved with increasing vehicle numbers, necessitating durable and sustainable materials. Since the 1980s, traditional road materials have been combined with unconventional ones [1]. Waste production is a major environmental concern, with industrial advancements generating vast amounts of trash annually [2]. Scientists have explored repurposing waste materials such as polymers, glass, steel slag, plastic, tires, and recycled concrete aggregates in road construction, benefiting environmental conservation and resource management [3]. Repurposing waste materials from landfills, including polymer, glass, steel slag, plastic, tires, aggregate dust, and recycled concrete aggregate, for pavement construction has significant advantages for environmental and resource conservation [4]. Consequently, the increasing use of recycled materials in asphalt pavements necessitates an environmental assessment of their effects on energy use and CO2 emissions, which can be achieved by reducing the energy consumption for preparing the mixtures [5]. With the serious hurdles posed by industrial enterprises in the civil engineering sector, the utilisation of recycled materials to ration the utilization of recycled materials to ration the use of natural resources in construction processes provides a potential solution [6]. Managing waste from demolished concrete structures is a global challenge [7]. However, asphalt mixed with recycled concrete aggregates often exhibits lower technical performance due to material quality concerns, particularly stripping potential [8]. Porcelain aggregates must meet standards for grain shape, frost resistance, and wear resistance when used in asphalt surfaces [9]. Recycled ceramic aggregates add more binder, filler, and air voids, but they don't change shape easily and keep their indirect tensile strength after being submerged [10]. When you add 5 % porcellanitic material to roller-compacted concrete, which is a zero-slump concrete that can be compacted by vibratory rollers [11], the compressive strength goes up. Using nonporous ceramic granite waste reduces bitumen use and enhances water resistance, compressive strength, and shear resistance.This makes the pavement last longer [12]. Researchers investigated the possibility of substituting natural aggregates in bituminous mixtures with recycled ceramic aggregates and porcelain tile waste. Adding them made the mixture have more binder, filler, and air holes. Additionally, it reduced the likelihood of deformation and maintained its indirect tensile strength even after submersion. Under good mechanical conditions, recycled ceramic aggregates can replace up to 30 % of natural aggregates and meet binder course performance requirements for medium- to low-traffic roads[10]. The addition of recycled ceramic aggregates improved binder course performance for medium- to low-traffic roads. These materials can replace up to 30 % of natural aggregates while maintaining mechanical properties. Using old porcelain tiles as recycled aggregate in concrete cleans up the environment, makes up for the lack of aggregate, and improves mechanical properties like bulk specific gravity, flow, stability, resistance to abrasion, and rutting [13]. Using leftover porcelain tile as a recycled aggregate also mitigates pollution and aggregate scarcity [14]. Additionally, recycled ceramic aggregates maintain tensile strength and resist plastic deformation [15]. Infrastructure widely recognises the values of sustainable construction and organic materials [16]. However, asphalt mixed with recycled concrete aggregates has lower technical performance than mixtures using natural aggregates [17]. Given the environmental impact of waste generation [18], incorporating C&D waste in pavement layers enhances rut resistance and increases OBC [19,20]. However, recycled concrete aggregates alone do not provide optimal results. This research investigated how using recycled concrete and porcelain waste together could make binder–asphalt cement mixtures stronger and last longer at different temperatures.

## Method details

### Materials

**Fig. 1:**
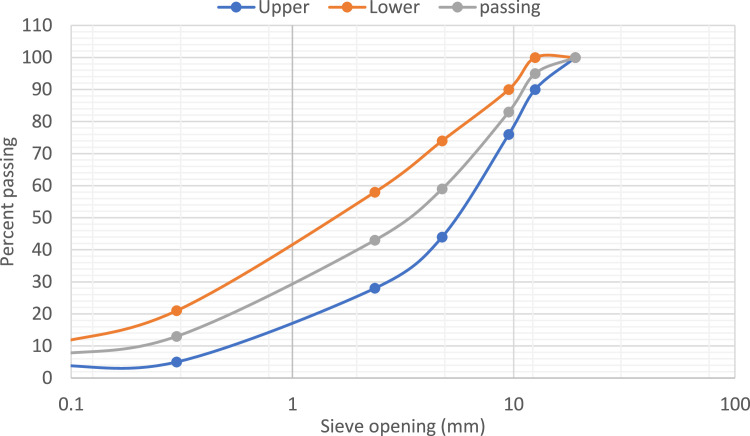
Gradation curve of the mixture aggregate per percent passing of the porcelain waste and recycled concrete at the upper, passing, and lower limits.

Asphalt grade 40–60, combined with concrete and porcelain waste from demolished residential buildings in Erbil City, were used in this study according to the aggregate graduation requirements for the asphalt binder course layer in ASTM D3531. Fig. 1 illustrates the gradation and percent passing of the aggregates for road projects to comply with the binder parameters of a common specification band [14].

Asphalt bitumen from Lanaz Refinery in Erbil, with a grade of 40–60, was used for the testing program. The conventional properties are summarized in Table 1.

**Table 1:** Conventional results of the asphalt binder test.

bitumen 40/60	Test method	Unit	Specification	Conformity
Penetration @ 25 °C	ASTM D5	mm/10	60–70	57
Softening point	ASTM D36	°C	49–56	52
Flash point	ASTM D92	°C	230 Min	280
Ductility	ASTM D113	cm	100 Min	130
Specific gravity @ 25 °C	ASTM D70	Kg/cm3	1.01–1.06	1.016

**Fig. 2:**
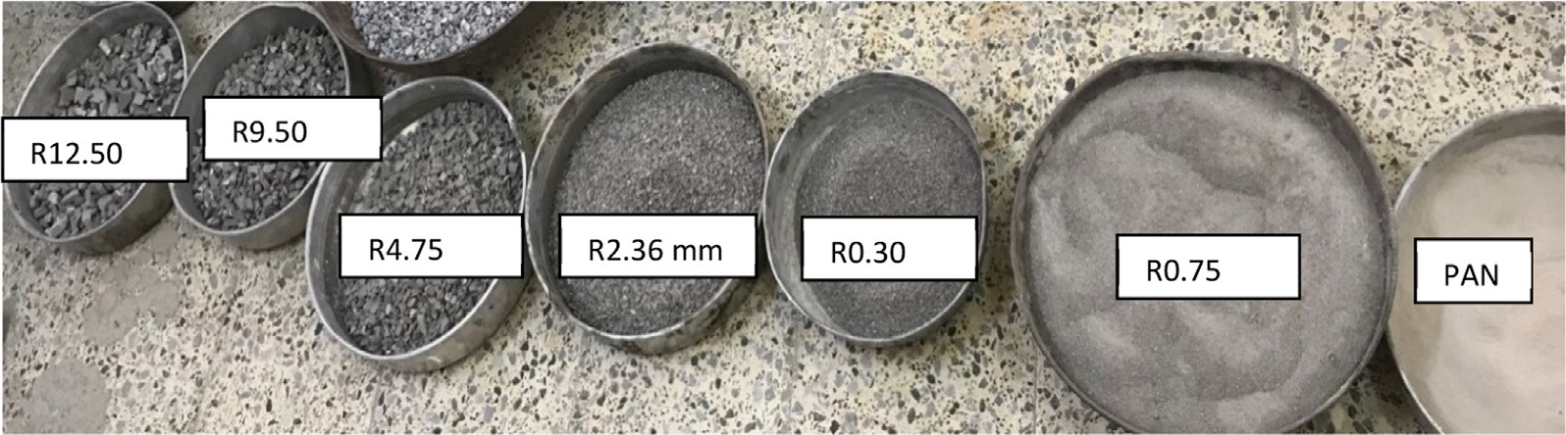
Gradation and particle shape of coarse, fine, and filler aggregates of porcelain waste. (R) refers to the particle size retained on the identified sieve number.

**Waste Porcelain:** Waste porcelain has the properties shown in Table 2 and shapes as in Fig. 2 from demolished buildings, made from Turkiye used in the testing program. The water absorption of waste porcelain at 0.57 % indicates that the material has low porosity and is relatively impermeable. This characteristic suggests that waste porcelain can be effectively used as a component in an asphalt cement mixture.

**Table 2:** Waste porcelain properties.

Porcelain properties	Test method	Unit	Conformity	Limits of Specification
Density	ASTM C127	Kg/m^3^	2380	2400–2700
Specific gravity	ASTM C127	-	2.27	2.3–2.6
Water absorption	ASTM C1585	%	0.57 %	-

**Fig. 3:**
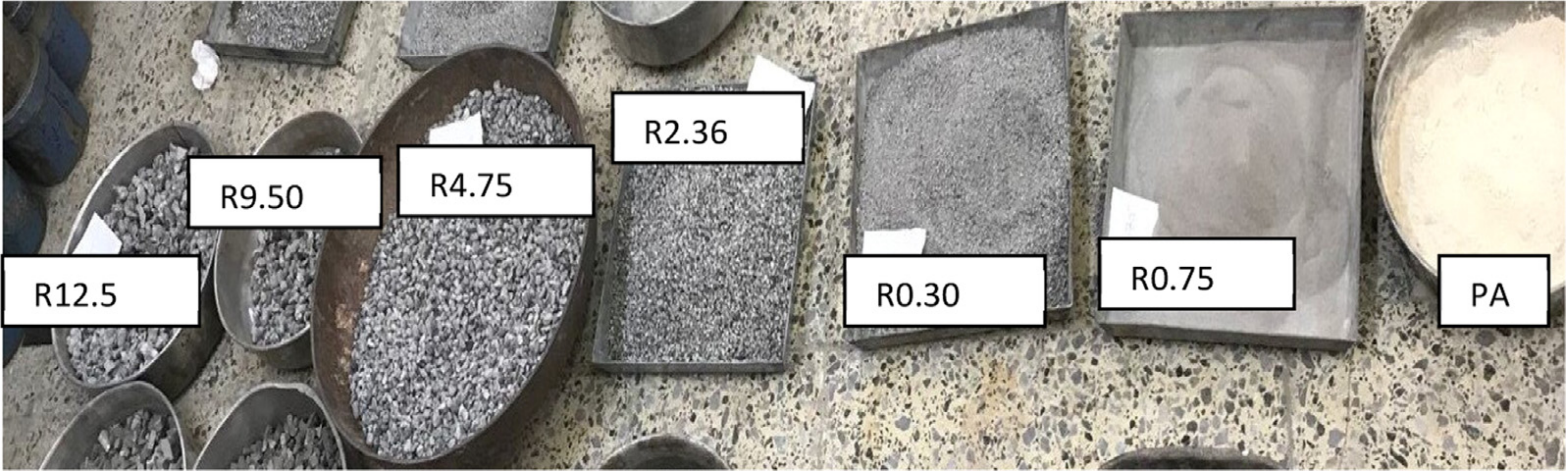
Gradation and particle shapes of the coarse, fine, and filler aggregates of recycled concrete waste. (R) refers to the particle size retained on the identified sieve number.

**Recycled Concrete Aggregate;** The concrete obtained from demolished buildings in Erbil city used particle shapes shown in Fig. 3, together with the characteristics enumerated in Table 3:

**Table 3:** Properties of the recycled concrete aggregate.

Aggregate Property	Test Code	Unit	conformity	Limits of Specification
Density	ASTM C127	Kg/m^3^	2480	2400–2700
Specific gravity	ASTM C127	-	2.506	2.3–2.6
Water absorption	ASTM C1585	%	5.34	6

### Testing types

**Fig. 4:**
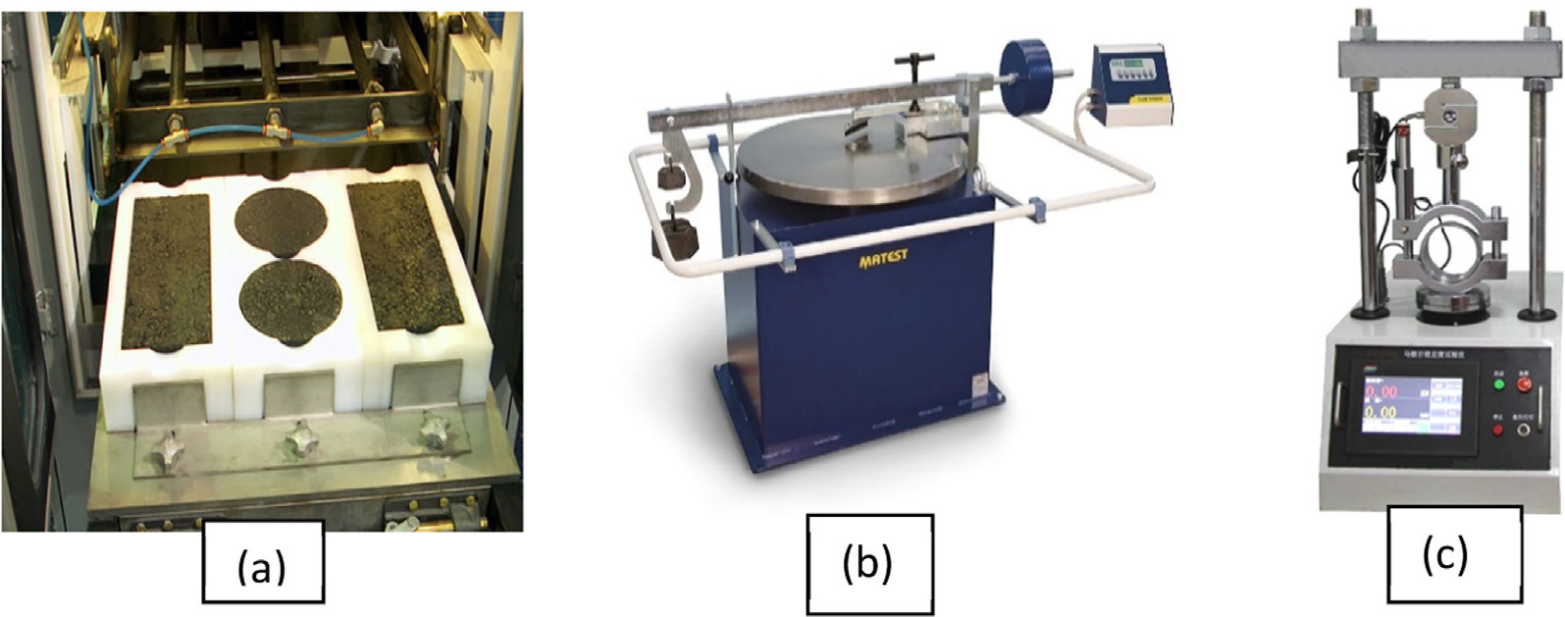
Test equipment: a) Hamburg wheel-truck device, b) Bohme abrasive test device, and c) Marshall test device.

The rutting test conducted following the Hamburg Wheel Tracking Test requirements as per AASHTO T 324–19, using two samples for each mixture, with half-circle beams 35 mm thick. Abrasion tests were performed using the Bohme abrasive wheel UTA-0615-T, with two samples of 70 × 70 × 100 mm for each mixture to evaluate the durability of the mixtures. The mechanical strength, flexibility, temperature susceptibility (TS), and index of retained strength (IRS) of the mixture were tested using the Marshall method as per ASTM D1559 and ASTM 6927–22, with three samples of 65 × 101 mm for each test. The shape and size of the devices used are shown in Figs. 4a–c, respectively.

Marshall tests were conducted at temperatures of 60 °C and 25 °C to determine the stability and flexibility, following the standard Marshall test procedures. Additionally, the temperature susceptibility (TS) and Index of Retained Strength (IRS) of the mixtures were evaluated according to ASTM D4123 and ASTM D1075/D1074, respectively. Below are four equations used to find out the (TS) and the (I.R.S.).

(1) $$ST\;=\frac{2P_{ult}}{\pi tD}\left(kg/cm2\right)$$

Where ST is tensile strength, Pult is ultimate load applied (kg), t is sample thinness (cm) and D is sample diameter in (cm).

(2) $$T.S.\;=\frac{S_{Ti}\;-S_{Tj}}{j-i}\left(kg/cm2/c^{\circ }\right)$$

Where T.S. is the temperature susceptibility, and STi and STj are the tensile strengths of the mixtures at *i* = 25 °C and *j* = 60 °C

(3) $$S=\;\frac{P_{ult}}{2\pi r^{2}}\left(kg/cm2\right)$$

where S is the compressive strength, Pult is ultimate load applied (kg), and r is radias (cm).

(4) $$I.R.S.\;=\frac{S_{2}}{S_{1}}*100>\;70\%$$

where S1 is the compressive strength of dry mixture tested at 25 °C and S2 is the compressive strength of conditioned mixture tested at 60 °C.

### Asphalt mixture proportioning and sampling

This study used an asphalt combination with the specific quantities of recycled concrete and porcelain aggregates listed in Table 4. Four types of Marshall mixtures were prepared with three replacement rates of porcelain (25 %, 40 %, and 60 %) for the recycled concrete aggregate for all mix gradations. Based on previous research [2,9, and 10] these percentages were chosen to show the impact of the replaced materials on the test results. Higher percentages signify better environment cleaning and finance saving while maintaining specifications according to standards. The samples were prepared according to ASTM D6927–22 for heavy-load highway (75 blows) Marshall testing, with three samples for each test.

**Table 4:** Weights of the recycled concrete and waste porcelain aggregates according to proportions of selected gradation of road binder layer.

Sieve Size (mm)	M1	M2		M3		M4	
	100 % Recycled concrete Agg. (g)	75 % Recycled concrete Agg. (g)	25 % Waste porcelain (g)	60 % Recycled concrete Agg. (g)	40 % Waste porcelain (g)	40 % recycled concrete Agg. (g)	60 % Waste porcelain (g)
19.000	0	0	0	0	0	0	0
12.500	222.3	166.73	55.58	133.38	88.92	88.92	133.38
9.500	108.3	81.23	27.08	64.98	43.32	43.32	64.98
4.750	199.5	149.63	49.88	119.70	79.80	79.80	119.70
2.360	176.7	132.53	44.18	106.02	70.68	70.68	106.02
0.300	262.2	196.65	65.55	157.32	104.88	104.88	157.32
0.075	91.2	68.40	22.80	54.72	36.48	36.48	54.72
PAN/filler	79.8	59.85	19.95	47.88	31.92	31.92	47.88

## Results and discussion

**Fig. 5:**
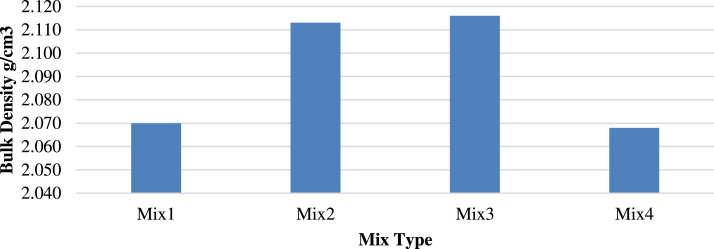
Density of different mixtures.

The average test results for the four types of mixtures are presented in Table 5. Porcelain is heavier than the replaced recycled concrete, as indicated by the increased density of the mixture with increasing rate of the porcelain replacement, except in M4. The decrease in the weight of M4 suggests that a further increase in the porcelain content forms gaps and voids in the material owing to its flat particle shape, as shown in Fig. 5.

**Table 5:** Mixture properties, code, and test results.

Properties	Specifications according to	Mix Code			
		M1	M2	M3	M4
Density (g/cm3)	AASHTO T 310	2.070	2.113	2.116	2.068
Bulk Sp. Gr. (Gmb)	AASHTO T209–94	2.066	2.108	2.102	2.086
Apparent Sp. Gr. (Gsb)	AASHTO T209–94	2.11	2.24	2.22	2.20
Stability at 60 °C (KN)	ASTM D6927–22	31.0	33.0	28.0	26.3
Marshall flow at 60 °C (mm)	ASTM D6927–22	3.6	4.6	4.2	4.4
Stability at 25 °C (kN)	ASTM D6927–22	52.0	50.3	49.0	47.3
Marshall flow at 25 °C (mm)	ASTM D6927–22	3.75	3.2	2.6	2.8

**Fig. 6:**
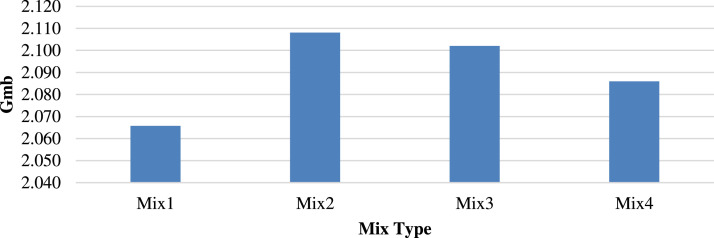
Bulk specific gravity (Gmb) of different mixtures.

**Fig. 7:**
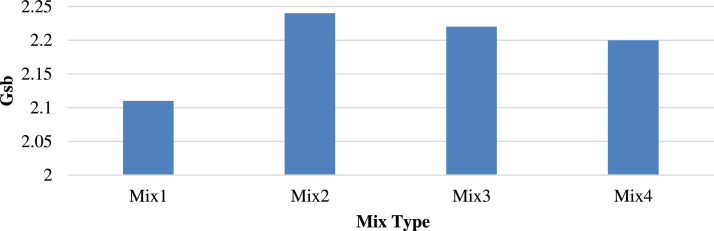
Apparent specific gravity (Gsb) of different mixtures.

Fig. 6, Fig. 7 show the Gmb and apparent specific gravity (Gsb), respectively, exhibiting the same trends. Replacing 25 % recycled aggregate with porcelain yielded the highest Gmb and Gsb values of 2.108 and 2.24, respectively, implying an increase of 2.00 % and 6.16 %, respectively.

**Fig. 8:**
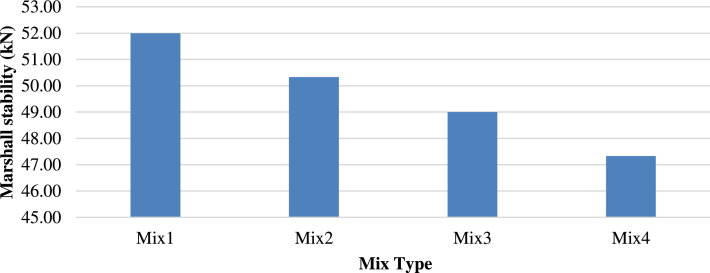
Marshall Stability of different mixtures tested at 25 °C.

**Fig. 9:**
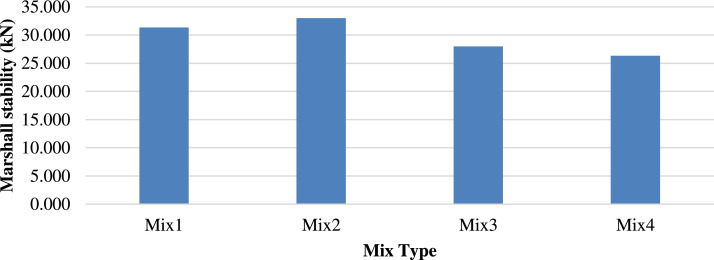
Marshall Stability of different mixtures tested at 60 °C.

Fig. 8, Fig. 9 show the Marshall stability of the mixtures at different temperatures The Marshall hardness decreased to 47.3 kN with the increased substitution of ceramic, reflecting a reduction of roughly 9 % at 25 °C, attributed to the greater stiffness of the porcelain compared to the concrete block particles. Meanwhile, M2 tested at 60 °C yielded the highest Marshall stability of 33 kN, exhibiting an increase of 6.4 % from that of M1. This indicates the increased hardness and stability of the porcelain particles.

**Fig. 10:**
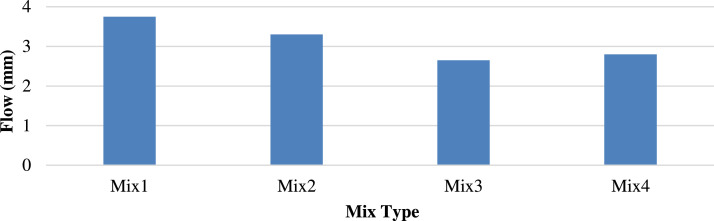
Marshall Flow of different mixtures at 25 °C.

**Fig. 11:**
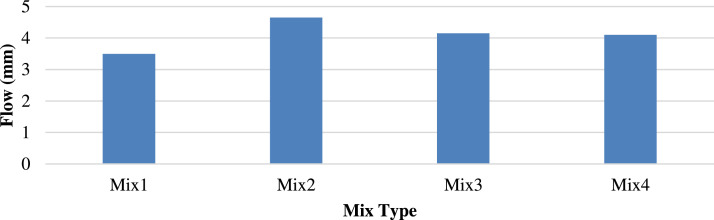
Marshall Flow of different mixtures at 60 °C.

Fig. 10, Fig. 11 show the different flow trends owing to the homogeneity of the materials, including the sample, particle shapes, and air gaps. The replacement of the recycled concrete aggregate with porcelain decreased the Marshall flow of M3 from 3.75 to 2.6 mm at 25 °C and increased the Marshall flow of M2 from 3.6 to 4.6 mm at 60 °C. This indicates the stability of porcelain particles at low temperatures.

**Fig. 12:**
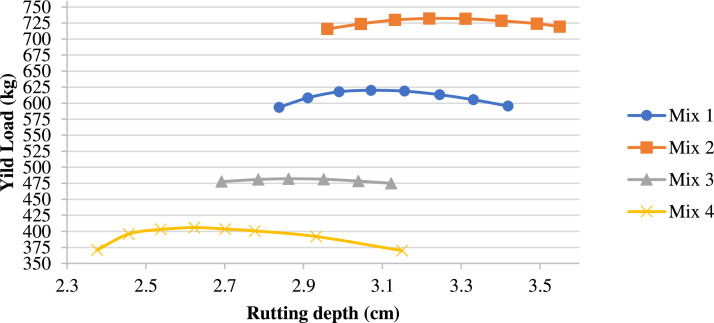
Rutting depth versus load applied for the mixtures.

The rutting deformation resistance of M2 improved by 22 %, as indicated by the bearing yild load, which is 732.1 kN. Meanwhile, the rutting depth of M2 only increased by 5 % compared to Mix 4, which has a bearing load of 406 kN and a rutting depth of 2.623 cm (Fig. 12). This suggests that porcelain addition should be limited based on the durability of the mixture. The recycled ceramic aggregates in hot mix asphalt, with a 20 % proportion, significant improvements in Marshall stability and resilient modulus strength, with a 25 % increment in Marshall stability and a 13.5 % increase in resilient modulus strength [21].

**Fig. 13:**
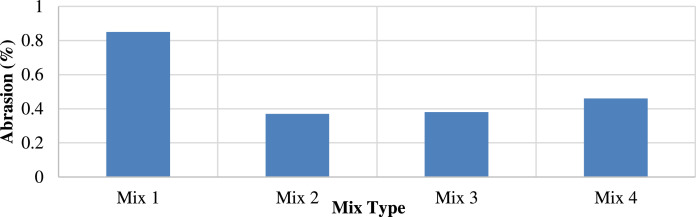
Abrasion resistance for all mixtures.

Abrasion improved with the addition of porcelain, as shown in Fig. 13. The highest abrasion resistance was obtained by adding 25 % porcelain to M2, denoting the high durability of the mixture.

**Fig. 14:**
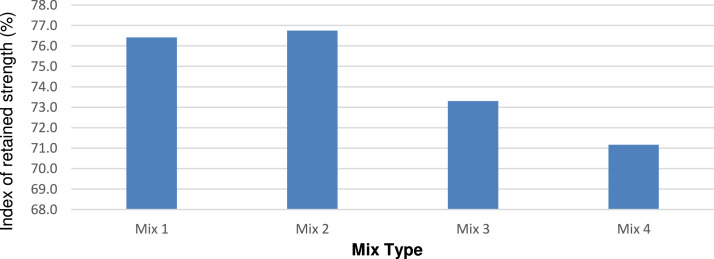
Index of retained strength for different mixtures.

The index of the retained strength was obtained according to ASTM d-1075 to determine the effect of the porcelain particles on the durability of the mixture. Mix 2 exhibited the highest external stress resistance and durability, as shown in Fig. 14.

**Fig. 15:**
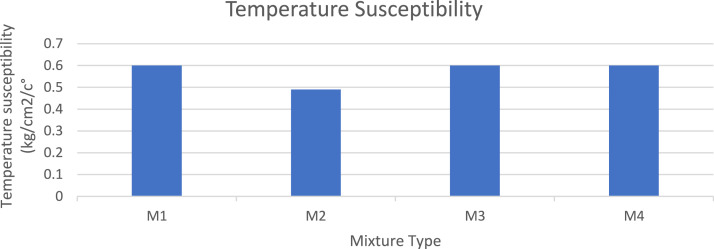
Temperature Susceptibility for different mixtures.

The mixtures temperature susceptibility has affected significantly at mixture M2, replacing porcelain affected negatively and make the mixture more susceptible, as show in Fig. 15.

### Method validation

The method was validated through a series of mechanical and durability tests, including wheel truck and Bohme abrasion tests while ensuring compliance with Marshall requirements. The results confirmed a 6 % increase in bulk specific gravity (2.24), an improvement in Marshall flow from 3.5 to 4.6 mm, and a 5 % rise in stability (33 kN). Additionally, the 25 % porcelain replacement exhibited the highest abrasion resistance and extended service life, though it increased susceptibility to temperature variations. These findings demonstrate the reliability and effectiveness of porcelain waste as a substitute for recycled concrete aggregates in asphalt mixtures, contributing to sustainable pavement solutions.

### Limitations

The described method does not have any known limitations under the specified experimental conditions and is applicable across all tested scenarios.

## Conclusion

The development of sustainable construction materials is a global goal. In highway construction, natural materials play a major role in producing durable and strong mixtures to achieve structures with long service life. The modification of these materials requires replacing, mixing, adding, and restrengthening their properties to produce stronger and more durable mixtures. This study focused on the use of ecofriendly materials by adding recycled concrete aggregate and replacing it with recycled porcelain to determine its effects on the mechanical properties and durability of asphalt–concrete binder mixtures. The main conclusions of this study are as follows:

1. The recycled concrete aggregate is a suitable and noticeably effective modifier for the asphalt binder mixture, yielding acceptable mechanical and durable results.
2. According to the test results, porcelain particles can replace recycled concrete aggregate at an optimal rate of 25 %.
3. Adding porcelain increased the density of the mixtures. Compared to M1, the bulk density of M3 increased by 2.2 %, whereas the Gmb of M2 increased by 2 %.
4. Replacing porcelain decreased the mechanical properties of the asphalt mixtures at low temperatures. However, the mechanical properties improved with the porcelain replacement rate of only 25 % in M2.
5. M2 improved the resistance of the asphalt mixture by 22 % for the rutting deformation, 20 % for the abrasion resistance, and 1 % for the index of retained strength. However, it negatively affected the temperature susceptibility of mixture M2, making it more temperature susceptible by 18 %.

## Recommendations

Highway construction is an ongoing and essential requirement, making the replacement primary components in highway mixtures with waste construction materials a sustainable and environmentally beneficial goal. This research demonstrates and recommends the utilization of waste concrete and porcelain aggregates in highway construction.

Further tests, particularly on the rheological properties of bitumen through bending beam rheometer and dynamic shear rheometer tests, are recommended to better understand the role of waste porcelain and recycled concrete aggregates as modifiers for pavement mixtures.

## CRediT authorship contribution statement

**Waleed A. Hamad:** Data curation, Funding acquisition, Resources, Software, Writing – original draft. **Ganjeena J. Khoshnaw:** Conceptualization, Supervision, Project administration, Investigation, Formal analysis, Writing – review & editing.

## Declaration of competing interest

The authors declare that they have no known competing financial interests or personal relationships that could have appeared to influence the work reported in this paper.
